# Leptomeningeal disease (LMD) after resection of brain metastases: results of the multicenter *SUBAROMA* study

**DOI:** 10.1007/s11060-026-05700-6

**Published:** 2026-07-10

**Authors:** Laura Mühlhausen, Martin Kocher, Hanah Hadice Karadachi, Ulrich Sure, Yahya Ahmadipour, Levin Häni, Danial Nasiri, Tommaso Araceli, Martin Proescholdt, Nils Ole Schmidt, Andrea Cattaneo, Vera Nickl, Florian Scheichel, Franz Marhold, Stefan J. Grau, Christina A. Hamisch, Franz L. Ricklefs, Yahya Zghaibeh, Roland H. Goldbrunner, Stephanie T. Jünger

**Affiliations:** 1 Center for Neurosurgery, Department of General Neurosurgery, Faculty of Medicine and University Hospital Cologne, University of Cologne, Cologne, Germany; 2 Center for Neurosurgery, Department of Stereotactic and Functional Neurosurgery, Faculty of Medicine and University Hospital Cologne, University of Cologne, Cologne, Germany; 3 Institute for Neuroscience and Medicine, Forschungszentrum Jülich, Jülich, Germany; 4 Department of Neurosurgery and Spine Surgery, University Hospital Essen, Essen, Germany; 5 Department of Neurosurgery, Inselspital, Bern University Hospital and University of Bern, Bern, Switzerland; 6 Department of Neurosurgery, Regensburg University Medical Center, Regensburg, Germany; 7 Wilhelm Sander Neuro-Oncology Unit, Regensburg University Medical Center, Regensburg, Germany; 8 Department of Neurosurgery, University Hospital Würzburg, Josef- Schneider-Str. 11, 97080 Würzburg, Germany; 9 Karl Landsteiner University of Health Sciences, Dr. Karl-Dorrek-Straße 30, Krems, 3500 Austria; 10 Division of Neurosurgery, University Hospital St. Poelten, Dunant-Platz 1, St. Pölten, 3100 Austria; 11 Klinikum Fulda, Academic Hospital of Marburg University, Fulda, Germany; 12 Department of Neurosurgery, Medical Center Hamburg Eppendorf, Hamburg, Germany; 13 Center for Integrated Oncology, Faculty of Medicine and University Hospital Cologne, University of Cologne, Cologne, Germany

**Keywords:** Neurosurgery, Brain metastases, Leptomeningeal disease, Resection, Systemic therapy

## Abstract

**Purpose:**

To identify risk factors for development of leptomeningeal disease (LMD) in patients with resected brain metastases (BM) based on the multicentric *SUBARoMA* study-cohort.

**Methods:**

The *SUBARoMA* study included patients undergoing surgery as initial treatment for BM. LMD was diagnosed based on MR imaging or lumbar puncture. Risk factors were identified from univariate Kaplan-Meier and multivariate Cox Regression analysis.

**Results:**

We analyzed 2,673 patients. In total, 148 developed LMD (11.1% of recurrences, 5.5% of all patients, 104 at first recurrence). Median time to LMD after surgery was 5.5 months (range: 0.2-129.3). Risk-factors for development of LMD were primary tumor type (PT) breast cancer and melanoma (*p* = 0.003), interval between BM and PT diagnosis ≥ 3 months, and incomplete resection (*p* = 0.002), while postoperative systemic therapy (*p* < 0.001) represented a protective factor. Incomplete resection (*p* = 0.003; HR 1.810; 95%CI 1.218–2.690) and postoperative systemic therapy (*p* = 0.028; HR 0.641; 95%CI 0.431–0.952) remained independent risk/ protective factors in multivariate analysis. Postoperative radiation-therapy and anatomical location did not relate to LMD-frequency. Over the study period, diagnosis of LMD, application of local radiation, targeted and immunotherapies increased while whole brain radiation decreased.

**Conclusion:**

Breast cancer, melanoma, incomplete resection and BM occurrence ≥ 3months after PT are risk-factors for LMD-development after BM resection, while postoperative systemic therapy represents a protective factor.

**Supplementary Information:**

The online version contains supplementary material available at 10.1007/s11060-026-05700-6.

## Key points

• Primary tumor type and systemic therapy may influence the development of LMD to greater extent than postoperative radiation therapy.

• Postoperative residual tumor potentially has a greater impact on LMD development than anatomic location.

• Gross total resection and application of postoperative systemic therapy, including immunotherapy and targeted therapy, reduce the risk of LMD development.

**Supplementary Information:**

The online version contains supplementary material available at 10.1007/s11060-026-05700-6.

## Introduction

Up to 30% of patients with systemic cancer develop brain metastases (BM) during the course of their disease [1]. Due to improved extracranial tumor control and advances in systemic, CNS-active, therapies, the prognosis of patients with BM has improved significantly [2]. Leptomeningeal disease (LMD) is a severe complication of BM and is defined as the dissemination of tumor cells along the leptomeninges and cerebrospinal fluid (CSF). LMD remains associated with a poor prognosis and reduced overall survival (OS) [3]. Due to improved OS of patients with BM and improved imaging techniques, an increasing incidence of LMD has been reported [4], highlighting the importance of a better understanding of this condition. Several clinical, anatomical, and treatment-related risk factors for the development of LMD in patients with BM have been identified. LMD most commonly occurs in patients with breast cancer, lung cancer, and melanoma [5]. A high overall disease burden, such as multiple BM and large tumor size, is an important predictor of LMD. An increased risk was also associated with anatomical BM location, e.g., BM near CSF compartments, direct ventricle contact, and infratentorial lesions [6]. Also, treatment-related factors can increase the risk of LMD, such as piecemeal resection and intraoperative access to the ventricles [7, 8]. Postoperative radiation therapy for BM using stereotactic radiosurgery (SRS) has been associated with a higher risk of LMD compared to whole-brain radiation therapy (WBRT); however, the latter is associated with cognitive side effects and inferior local control [9].

The existing literature regarding predictive factors for LMD is heterogeneous due to small sample sizes, retrospective observations, and studies limited to a specific primary tumor type. Therefore, the development of general recommendations for avoiding and treating LMD has been challenging, and there still is an ongoing need to better characterize the risk factors of LMD development in order to improve therapeutic decision-making. The aim of this trial, as part of the *SUBARoMA* study (Surgery for brain metastases: a retrospective multicenter analysis), is to identify risk factors for postoperative LMD development based on a large multicentric cohort of BM patients who were surgically treated between 2012 and 2022.

## Methods

### Study design

We conducted a multicenter, retrospective cohort study of patients with BM from eight neurosurgical departments in Germany, Austria, and Switzerland in order to analyze therapeutic interventions and oncological outcomes in patients operated for at least one BM. The first analysis was previously published [10]. This paper will focus on patients developing LMD after BM resection and identify the underlying risk factors.

### Patients

Each center reviewed its internal database to include patients according to the following inclusion criteria:

i) no prior local treatment of BM before surgery.
ii) initial surgery for BM between 2012 and 2022.
iii) age ≥ 18 years at the time of BM surgery.

Indications for surgery, except for emergencies, were made by interdisciplinary institutional tumor boards. Dominant reasons for surgery were neurological symptoms caused by the BM and diagnostic confirmation [10].

### Data acquisition and definition of parameters

Demographic, clinical, treatment, and outcome parameters were retrieved from the electronic patient records and recorded by the individual centers in a standardized data sheet. The data was then anonymized, centrally reviewed by SJ and MK, and merged into a single database [10]. The interval to BM diagnosis was defined as the time between the diagnosis of the primary tumor (PT) and the date of BM resection and defined as i) BM diagnosis before or ≤ 3 months after PT diagnosis, ii) > 3 months after PT diagnosis [11, 12]. The Karnofsky Performance Status (KPS) of patients was assessed pre- and postoperatively. Surgery was performed using state-of-the-art techniques such as microsurgery, optical neuro-navigation, intraoperative ultrasound, pre-/ intraoperative electrophysiological brain mapping/ monitoring, and fluorescence guidance. The extent of resection (EOR) was assessed based on the radiologic reports of postoperative contrast-enhanced magnetic resonance imaging (MRI) and classified as follows:

i) gross total (GTR) – no residual tumor.
ii) near total (NTR) – no nodular residual tumor but linear contrast enhancement.
iii) subtotal (STR) – evidence of nodular residual tumor.

Radiological and clinical follow-up were performed according to institutional policies and included regular clinical assessment and MRI. Standardized follow-up protocols were only introduced for some PT [13], therefore an overarching standardized follow-up procedure was not granted for most of the study-period. The following types of brain recurrence were defined and diagnosed from the follow-up MR images:

1. local intracranial recurrence: at resection cavity only.
2. distant intracranial recurrence: no contact to the resection cavity.
3. combined intracranial recurrence: combination of local and distant brain recurrence.
4. leptomeningeal disease (LMD): diagnosed by MRI and/ or CSF sampling.

According to institutional protocols, different types of postoperative radiotherapy were applied. Systemic therapy was continued or initiated depending on extracranial tumor manifestation, molecular diagnostics and available therapeutic options.

### Statistical analysis

The statistical analysis was performed using SPSS software (version 29.0; IBM, Armonk, NY, USA). P-values < 0.05 were considered statistically significant. Overall survival and time to any type of intracranial failure including LMD were calculated from the date of BM resection using the Kaplan-Meier method. For the present analysis, diagnosis of LMD represented the primary endpoint. For the analysis of OS, the date of death was recorded and patients known to be alive were censored at their last follow-up, but no later than the database closure date (31st of March 2023). Post-LMD survival was calculated from the date of LMD diagnosis to the date of death or last follow-up. For the analysis of time to LMD, patients who were not diagnosed with LMD were censored either at the time of last follow-up or at the time of death.

Factors with a significant influence on the development of LMD were determined by univariate Kaplan-Meier analysis using the log-rank test. To identify independent predictors of LMD recurrence, parameters with known clinical and prognostic significance were entered into a multivariate Cox regression model. For Kaplan-Meier analysis, categorical and continuous variables were divided into two or more appropriate groups, and statistical significance for the complete models was computed. Before entering the Cox-regression model, all variables were dichotomized, and the hazard rates for the variables finally kept in the model were calculated. Only patients with the respective available data for the individual factors were included in uni- and multivariate survival analyses.

## Results

### Patient acquisition

The eight participating neurosurgical departments (6 German, 1 Swiss, 1 Austrian) collected data from a total of *n* = 3,203 patients. Of these, 530 patients were excluded due to date of the first BM resection not matching the defined period, less frequently, incorrect primary tumor diagnosis or LMD present at first diagnosis.

### Patient and treatment characteristics

The gender distribution of the overall cohort was balanced (50.2% women), and the median age at the time of resection was 63.2 years (range 19.4–87.8). The most common PT entities were non-small cell lung cancer (NSCLC, 42.5%), breast cancer (12.0%), and malignant melanoma (10.7%). Treatment consisted of surgery, radiation therapy, systemic therapy or a combination. More than two thirds of patients had active extracranial tumor at the time of surgery. Most patients presented with neurological symptoms and were diagnosed with BM > 3 months after PT diagnosis (58.3%). The majority was operated on one BM (92.3%). Extent of resection was known in 1,738 patients and classified as GTR in 72.7%. Due to individual institutional practice and missing postoperative MRI, in 935 patients (35.0%) assessment of EOR was not possible. To rule out potential bias, a Kaplan Meier analysis comparing patients with and without postoperative imaging-based assessment of EOR with respect to LMD development was carried out that did not reveal a statistically significant difference (*p* = 0.175).

After surgery, general performance measured by KPS improved significantly (*p* < 0.001). Following surgery, 58.1% of patients underwent postoperative systemic and 78.9% postoperative radiation therapy (Table 1). Of patients undergoing systemic therapy, 62.0% received targeted- and/or immunotherapies, however application frequencies varied across primary tumor types, and accounted for 40.4% in breast cancer, 40.5% in NSCLC, and 68.9% in melanoma patients. Amongst patients developing LMD, 42.2% received targeted- and/or immunotherapy. For further details of postoperative therapy including center and PT specificities please refer to our previous publication [10].

**Table 1:** Patient and treatment characteristics

Parameter		Total *n* = 2673	LMD *n* = 148	Percentage All /LMD
Gender	female	1343	74	50.2/50.0
	male	1330	74	49.8/50.0
Age	median	63.2 years	61.6 years	
	range	19.4–87.8	26.89–80.81	
	> 65	1149	57	43.0/38.5
Primary tumor (PT)	Non-Small Cell Lung Cancer	1136	45	42.5/30.4
	Breast cancer	322	34	12.0/23.0
	Malignant Melanoma	287	21	10.7/14.2
	Colorectal carcinoma	200	11	7.5/7.4
	Small cell lung cancer	145	5	5.4/3.4
	Cancer of unknown primary tumor	102	7	3.8/4.7
	Renal Cell Carcinoma	100	1	3.7/0.7
	Oesophagus carcinoma	75	3	2.8/2.0
	Urothelial carcinoma	55	3	2.1/2.0
	Prostate cancer	45	2	1.7/1.4
	Other entities	208	16	7.8/10.8
Operation PT	no yes n.a.	1247 1394 32	47 101	47.2/31.8 52.8/68.2
Radiation therapy PT	no yes n.a.	1739 873 61	75 71 2	66.6/52.4 33.4/48.6
Systemic therapy PT	systemic therapy exclusive targeted and immunotherapy systemic therapy including targeted and/or immunotherapy none n.a.	891 932 793 57	50 60 36 2	31.3/34.2 35.6/41.1 30.3/24.7
Any therapy PT	no yes n.a.	496 2143 34	19 129	18.8/12.8 81.2/87.2
Time interval between PT & BM	median	10.4 months	16.6	
	range	− 60.6–476.4	-8.52–313.8	
	before/ <3 months after primary tumor	1115	40	41.7/27.0
	> 3 months after primary tumor	1558	108	58.3/73.0
Active extracranial tumor at diagnosis of BM*	no	712	46	28.4/31.7
	yes	1798	99	71.6/68.3
	n.a.	163	3	
Number of BM	median	1	1	
	range	1–41	1–30	
	1 BM	1662	95	62.2/64.2
	> 1 BM	1011	53	37.8/35.8
Number of resected BM	1	2467	137	92.3/92.6
	> 1 BM	206	11	7.7/7.4
Extent of resection	gross total resection	1263	69	72.7/62.2
	near total resection	275	27	15.8/24.3
	subtotal resection	200	15	11.5/13.5
	n.a.	935	37	
KPS preoperative	median	80	80	
	range	20–100	20–100	
	≥ 70 < 70	2258 415	129 19	84.5/87.2 15.5/12.8
KPS postoperative	median	80	90	
	range	0–100	30–100	
	≥ 70 < 70	2314 359	132 16	86.6/89.2 13.4/10.8
Radiation therapy after BM resection	none	471	21	18.2/14.2
	whole brain radiation therapy	481	20	18.6/13.5
	focal radiation (radiosurgery, fractionated radiotherapy)	1629	107	63.2/72.3
	n.a.	92		
Systemic therapy after BM resection	systemic therapy exclusive targeted and immunotherapy	595	24	23.3/16.3
	systemic therapy including targeted and/or immunotherapy	959	62	37.6/42.2
	none	997	24	39.1/16.3
	n.a.	122	1	

No., number; BM, brain metastasis; PT, primary tumor; KPS, Karnofsky performance status; n.a., not available. * Progressive primary tumor and/or systemic metastases

### Leptomeningeal recurrence

Within the observation period and a mean imaging follow-up of 11.5 months (median: 4.3 months; range 0-123.6 months), 998 patients experienced a total of 1,328 recurrences, most of which occurred at the site of initial resection 505 (38.0%) and/or at distant location 675 (50.8%). A total of 148 patients were diagnosed with LMD. Hence, LMD accounted for 11.1% of all recurrences and in total affected 5.5% of all patients included in the analysis. In 104 (70.3%) patients, LMD occurred at first recurrence of intracranial disease. The occurrence of LMD was associated with the development of additional recurrences at the site of initial BM resection or at distant location (*p* < 0.001).

**Fig. 1:**
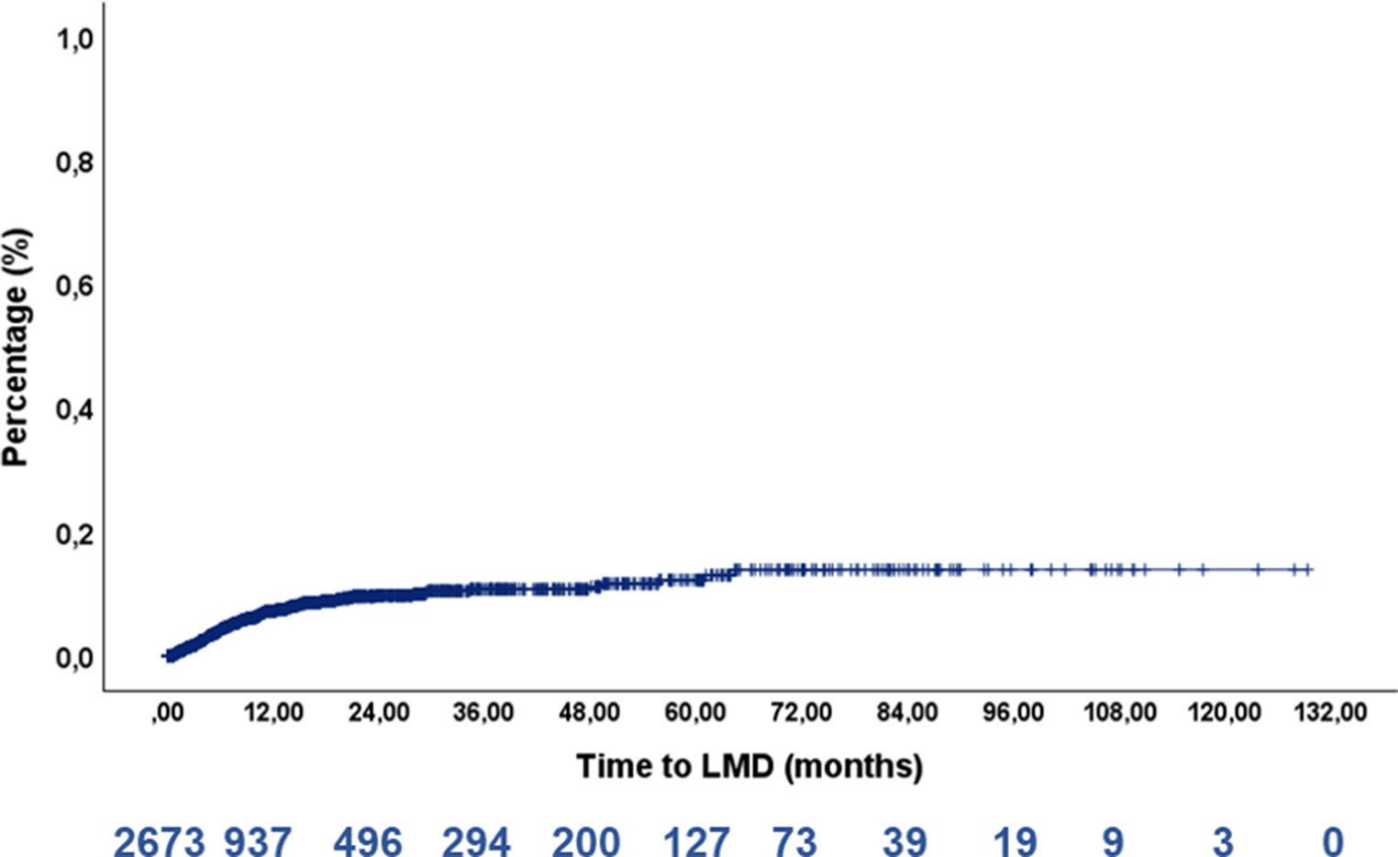
Kaplan-Meier-analyses for risk of development of LMD. LMD, leptomeningeal disease

**Fig. 2:**
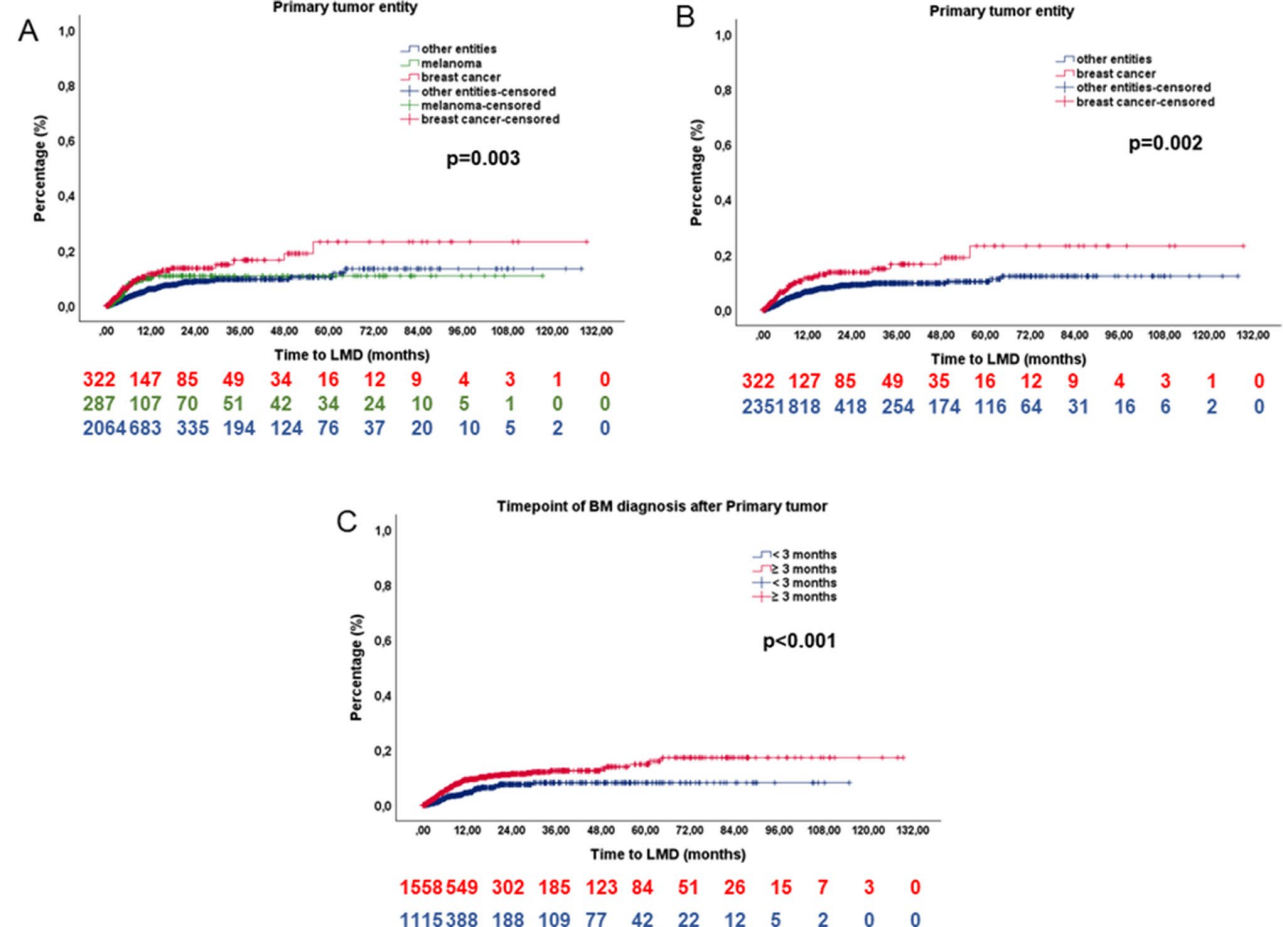
Kaplan-Meier-Analyses for risk factors for LMD progression; the proportion of patients developing LMD is shown in all graphs. (**A**), (**B**): Impact of primary tumor entity; (**C**): Impact of timepoint of BM diagnosis. LMD, leptomeningeal disease; BM, brain metastasis

The actuarial risk of LMD recurrence was 4.3% after 6 months and increased to 7.4% after 12 and to 9.8% after 24 months (Fig. 1). For patients who actually experienced LMD, the estimated median time to LMD was 5.52 months (range: 0.2-129.31 months). In the univariate analysis, PT entity (*p* = 0.003) and timepoint of BM development (*p* < 0.001) represented significant risk-factors for development of LMD. Breast cancer inherited the highest and melanoma the second highest risk, and the risk for LMD was higher for BM occurring later than 3 months after diagnosis of the PT (Fig. 2; Table 2). Active extracranial tumor at BM diagnosis, postoperative KPS, number of total/ resected BM (Table 2) and cerebellar location of the resected BM did not present significant risk factors for LMD.

**Table 2:** Univariate Kaplan Meier and multivariate cox regression analysis for development of LMD

Parameter	Development of LMD	
	univariate Kaplan Meier	multivariate Cox Regression (*n* = 1495)
Gender (female vs. male)	0.543	**-**
Age (< 65years vs. >65 years)	0.846	**-**
*Primary tumor (PT)* Breast cancer vs. Melanoma vs. Rest	0.010 0.003	
*Breast cancer vs. Rest*	0.002	n.s.
Therapy PT any vs. none	0.426	**-**
*Time interval between PT and BM* ≥ vs. < 3 months	< 0.001	n.s.
Active extracranial tumor at BM diagnosis (yes vs. no)	0.078	n.s.
Number of BM (> 1 vs.1)	0.998	n.s.
No. of resected BM (1 vs. >1)	0.874	**-**
*Extent of Resection*		
NTR + STR vs. GTR	0.002	0.003 (HR 1.810; 95%CI 1.218 − 2.690)
KPS preoperative (≥ 70 vs. <70)	0.708	**-**
KPS postoperative (≥ 70 vs. <70)	0.139	n.s.
*Radiation therapy after BM resection*		
WBRT vs. local RT vs. none any vs. none WBRT vs. local RT	0.274 0.192 0.358	n.s.
*Systemic therapy after BM resection*		
With IT/TT vs. without IT/TT vs. none any vs. none	0.03 < 0.001	0.028 (HR 0.641; 95%CI 0.431 − 0.952)

**-**: not included in the model

EOR, Extent of Resection; GTR, gross-total resection; NTR, near-total resection; STR, sub-total resection. No., number; HR, Hazard Ratio; CI, Confidence Interval; BM, brain metastasis; PT, primary tumor; KPS, Karnofsky Performance Status. WBRT, whole brain radiation therapy; RT, radiation therapy; IT, immunotherapy; TT, targeted therapy

**Fig. 3:**
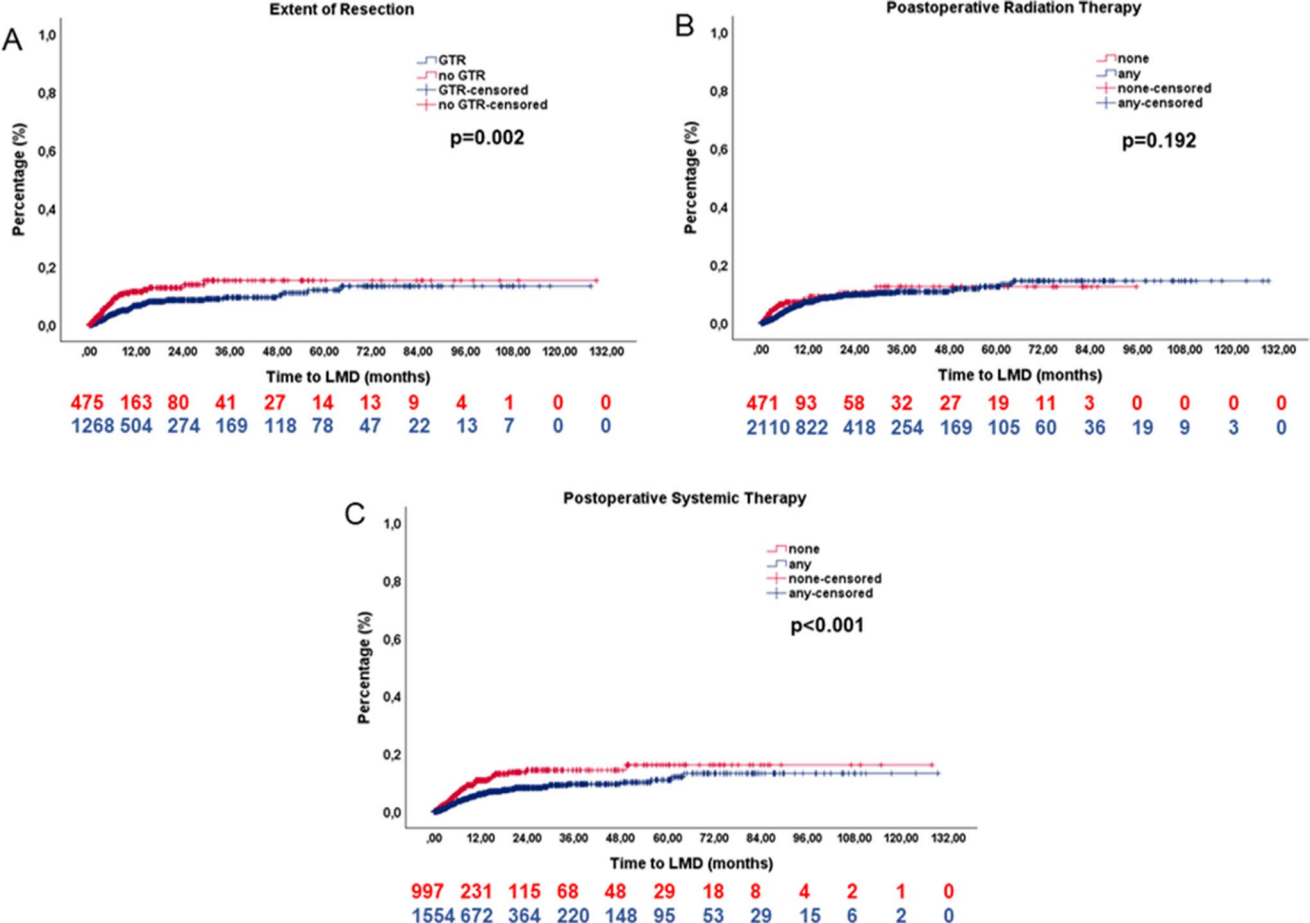
Kaplan-Meier-Analyses for risk factors for LMD progression associated with therapy: (**A**) Extent of resection, (**B**) radiation therapy, (**C**) systemic therapy. LMD, leptomeningeal disease; GTR, gross-total resection

Regarding therapy, GTR (*p* = 0.002) and application of postoperative systemic therapy (*p* < 0.001 represented significant protective factors against LMD. A separate analysis showed that both the application of targeted/immunotherapy and classic chemotherapy had a protective effect (*p* = 0.03, Supplement 1), while postoperative radiation therapy had no significant impact (Fig. 3). Also, the type of postoperative irradiation (WBRT versus local radiation therapy, *p* = 0.358, Supplement 1) did not significantly influence the development of LMD. In the multivariate Cox regression analysis including the significant variables from the univariate analysis and a list of other clinically relevant factors, incomplete resection (*p* = 0.003; HR 1.810; 95%CI 1.218–2.690) and postoperative systemic therapy (*p* = 0.028; HR 0.641; 95%CI 0.431–0.952) remained independent risk/ protective factors (Table 2).

### Overall and post-LMD survival

Median and mean OS of the total cohort were 7.5 and 15.0 months (range: 0.0-129.3 months). At the time of analysis, after a median follow-up of 8.5 months, 1,596 (59.7%) patients had died. In most patients, the cause of death was unknown (62.0%); in the remaining 606 patients, the most common cause of death was systemic progression (63.0%), followed by intracranial recurrence (32.0%), and other causes (5.0%). Median and mean post-LMD survival were 1.8 and 4.4 months, respectively (range: 0.1–69.9 months), and 121 of 148 (81.8%) patients with LMD died within the study period. In patients with LMD and known cause of death (*n* = 64), intracranial progression represented the most frequent event (*n* = 41, 64.1%).

### LMD and treatment over time

**Fig. 4:**
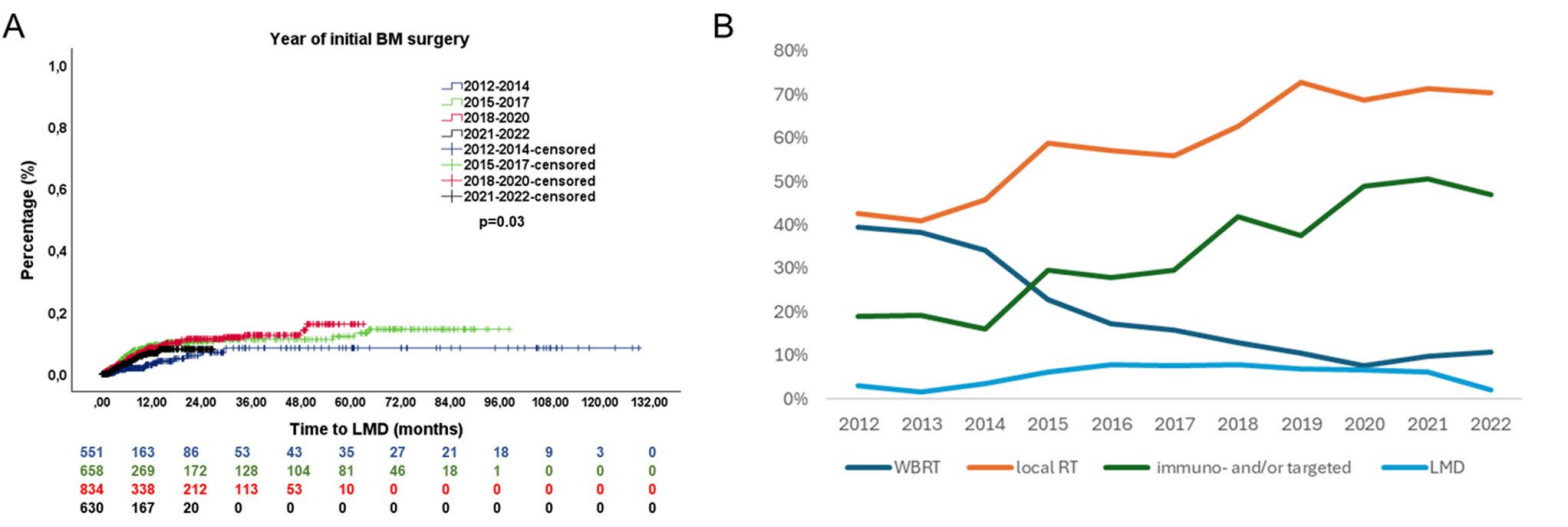
Changes of LMD rates and treatment modalities between 2012 and 2022 with (**A**) Kaplan-Meier-Analyses with time groups demonstrating differences in LMD development after brain metastasis (BM) resection and (**B**) change of individual parameters over time. LMD, leptomeningeal disease; RT, radiation therapy; WBRT, Whole brain radiation therapy

Comparing the frequencies of LMD diagnosis within the study period of 11 years a moderate increase becomes evident (Fig. 4A, B). LMD was diagnosed more frequently, especially between 2012 and 2016 with an increase from 3% in 2012 to 7.9% in 2018 and remained stable afterwards (Fig. 2B). At the same time the absolute number of patients as well as OS increased (Fig. 4A, B). The frequency of postoperative WBRT decreased from 40% in 2012 to 11% in 2022, while focal radiation therapy (FRT) increased from 43% in 2012 to 70% in 2022. The same pattern applied for the use of targeted and/ or immunotherapies (19% in 2012, 47% in 2022).

## Discussion

In this large multicenter cohort of 2,673 patients undergoing primary surgical resection of BM, LMD occurred in 5.5% of patients and accounted for 11.1% of all intracranial recurrences. The actuarial 24-month LMD risk of 9.8% confirms that LMD remains an uncommon event following BM resection. This rate of LMD diagnosis is consistent with contemporary surgical series, reporting rates between 11% and 22%, depending on PT entities and follow-up periods [14, 15], however, due to a median imaging follow-up of 4.3 months potential under detection-bias needs to be considered.

Despite its relative rarity, LMD is associated with a disproportionate clinical impact. The observed median post-LMD survival of 1.8 months is even shorter than reported in several recent melanoma or breast cancer series [16]. However, it should be noted that these findings were obtained under controlled study conditions and may therefore not be directly generalizable to real-world clinical settings. Intracranial progression predominated as cause of death (64.1%) in patients with LMD, reinforcing LMD as a terminal event, while in the present cohort, systemic progression represented the most frequent cause of death, in line with the literature [17]. The increase in LMD diagnosis between 2012 and 2016 likely reflect prolonged OS rather than true biological escalation. As systemic therapies improve extracranial tumor control, patients may live long enough to develop LMD as previously described in melanoma and HER2-positive breast cancer cohorts [18]. Improved MRI resolution and increased awareness may also contribute to the observed higher incident rates.

### Tumor biology, the key driver of LMD

In the present study, primary tumor entity was identified as a major determinant of LMD risk. Breast cancer conferred the highest risk, followed by melanoma. These findings are biologically plausible and consistent with prior literature [1, 19]. Breast cancer, particularly HER2-positive and triple-negative subtypes, has long been associated with leptomeningeal spread [18], due to a specific tropism in tumor subtype supporting spread to leptomeninges [20]. This observation may also be explained due to prolonged survival of these patients with modern HER2-targeted therapies and enhanced CNS surveillance of this subgroup. Tewarie et al. demonstrated entity-specific differences in LMD risk across BM patients in a meta-analysis, highlighting breast cancer as a high-risk group [19]. Our findings corroborate this observation in a large surgical cohort. Melanoma showed the second-highest risk of early LMD development, indicating rapid leptomeningeal progression in susceptible patients. Melanoma is characterized by aggressive CNS invasion and hematogenous dissemination. Although immune checkpoint inhibition and targeted therapy have dramatically improved survival in melanoma patients, leptomeningeal involvement remains associated with rapid deterioration, also observed in our cohort [21].

However, potential bias due to therapeutic options differing between individual primary tumor entities and molecular subgroups, as well as variations in imaging and clinical follow-up intervals during the long study period in the absence of uniform guidelines may have influenced frequencies of LMD diagnosis and cannot be ruled out in this study.

### Surgical considerations

Contrary to prior surgical assumptions, cerebellar location was not associated with increased LMD risk in our cohort. Historically, infratentorial tumors were considered at higher risk due to proximity to the fourth ventricle and basal cisterns, theoretically facilitating CSF dissemination [6]. Our data suggest that anatomical location alone does not drive leptomeningeal spread. However, the reported risk factors of ventricular entry and CSF exposure [22], was as well as size or configuration of BM were not covered in our study protocol and thus warrants further studies.

Gross total resection was associated with reduced rates of LMD diagnosis. This finding supports the principle of maximal safe cytoreduction [23]. Piecemeal, compared to en bloc, resection has been linked to higher risk of LMD [24], the same applies for the overall risk of local recurrence [25]. Yet, surgical technique, such as piecemeal vs. en bloc resection was not addressed by our protocol in detail.

Another critical point is the proportion of patients where determination of EOR was not possible, however, potential bias regarding LMD development was ruled out by a subgroup analysis.

Overall, the association between GTR and improved outcomes may partially reflect a less invasive tumor biology, although this relationship may additionally be confounded by anatomical and other selection factors [26]. The importance of GTR has also been underlined by a microscopic evaluation of resection margins, which showed no tumor cells infiltrating the surrounding brain tissue [27]. Residual tumor cells that persist after subtotal or near total resection may gain secondary access to CSF pathways and contribute to LMD development. Notably, Hulsbergen et al. demonstrated worse OS and PFS and higher occurrence of LMD in patients with residual tumor on postoperative MRI after BM resection [23].

### Radiation therapy

Historically, WBRT was assumed to sterilize microscopic disease, potentially reducing leptomeningeal spread when compared to FRT [28]. However, our data do not support this hypothesis. Over the study period, the use of WBRT declined substantially, while FRT increased. Focal radiation therapy, e.g. cavity-based SRS, serves several advantages when compared to WBRT, e.g. preserving cognitive function while OS remains uncompromised. Especially after 2014, LMD diagnosis increased while WBRT frequency has decreased as first line treatment. If prohibitive doses of radiation have been applied to organs at risk in the first course or in the case of disseminated intracranial progression, retreatment with WBRT serves as salvage treatment [29]. Neither, comparing WBRT and FRT nor any radiation therapy vs. none revealed a significant impact on LMD development. The question whether WBRT may prevent LMD development in some patients disregarding the tumor biology may not be answered by our data to full extent. Overall, it needs to be emphasized that neither exact time, radiation dose nor extension of radiation fields were covered by our study protocol, consequently, to draw definite conclusions considering the impact of postoperative radiation therapy, further investigations are needed. Considering the preservation of cognitive function by postoperative stereotactic radiosurgery without compromising OS [30] as well as the low overall incidence of LMD weakens arguments for routine use of WBRT to prevent dissemination.

### Systemic therapy

Postoperative systemic therapy, particularly targeted and/or immunotherapy, was associated with reduced LMD risk and improved survival. This observation is supported by Glitza Oliva et al. reported durable responses in melanoma LMD under immune checkpoint blockade, suggesting that systemic therapy can exert clinically relevant effects within the leptomeningeal compartment [16]. Our data indicate that targeted/ immunotherapy alone or in combination with classic cytotoxic chemotherapy are superior to classical systemic approaches with respect to OS. The use of targeted and immunotherapy has steadily increased since 2014 and is now recommended in international guidelines for the management of brain metastases [31, 32]. The results of our data support the view that systemic disease control, and a potential direct CNS activity, plays a central role in preventing or delaying LMD and that systemic therapy may exert a greater impact on LMD risk modulation. However, the aspect of higher treatment dedication and follow-up intensities, needs always be considered when interpreting survival data.

### Limitations

A central limitation of this study is that the dataset was not originally designed to investigate LMD as a primary endpoint, leading to LMD diagnosis being based on MRI and/or CSF cytology, without distinction between radiographic-only and cytologically confirmed cases.

Furthermore, the initial study evaluated predictors of local intracranial recurrence and OS in a multicentric, real-world cohort of patients with BM, undergoing surgery as first local therapy. Consequently, surgical parameters potentially associated with leptomeningeal spread, e.g. ventricular opening, or en-bloc vs. piecemeal resection as well as detailed anatomic specificities, e.g. proximity to CSF spaces, configuration or size of BM were not systematically recorded with respect to the literature, the absence of these variables limits mechanistic interpretation.

In addition, postoperative imaging follow-up was not standardized across centers. During the study period (2012–2022), regular postoperative MRI and uniform 3-monthly MRI surveillance, which is now recommended in neuro-oncological guidelines, was not consistently implemented in all participating centers, owed to the fact that the respective guidelines were only published in 2021/ 2022. We compared patients with and without postoperative MRI with regard to LMD development to exclude this bias. With a median imaging follow-up of 4.3 months, asymptomatic LMD may still have been under-detected, though.

Furthermore, despite the relevance of tumor biology, owed to the study period, molecular markers were not assessed to sufficient extent to draw conclusions. The same applies for detailed information regarding radiation therapy, including exact postoperative onset, dose, fractionation and extent of the radiation field.

Prospective studies incorporating standardized imaging intervals, molecular profiling, and detailed surgical variables are warranted.

## Conclusion

In this large multicenter surgical cohort, leptomeningeal disease occurred in approximately 5.5% of patients and was associated with extremely poor prognosis. LMD risk was associated with tumor biology, gross total resection, and application of systemic therapy, including targeted and immunotherapy, rather than with anatomic location or postoperative radiation therapy. In the era of prolonged survival through advances due to systemic treatment and more dedicated patient follow-up, LMD may become proportionally more visible, emphasizing the importance to develop preventive strategies, including optimized cytoreduction and CNS-active systemic therapy.

### Lay summary

After surgery for brain metastases, some patients develop a serious condition where cancer spreads to the sheaths around the brain and spinal cord (leptomeninges). This study analyzed over 2,600 patients with brain metastases to identify risk factors for development of leptomeningeal disease. About 6% developed this complication, usually within seven months, with reduced overall survival. Higher risk was linked to certain cancer types, especially breast cancer and melanoma; incomplete tumor removal, and later occurrence of brain metastases after diagnosis of the primary tumor. These findings may help identify high-risk patients earlier and improve monitoring and treatment decisions.

## Supplementary Information

Below is the link to the electronic supplementary material.

Supplementary Material 1

Supplementary Material 2

## Data Availability

Underlying patient data is available from the corresponding author on reasonable request.

## Abbreviations

BM: Brain Metastasis
CSF: Cerebrospinal fluid
CI: Confidence Interval Events
EOR: Extent of resection
FRT: Focal radiation therapy
GTR: Gross total resection
HR: Hazard Ratio
KPS: Karnofsky performance status
LMD: Leptomeningeal disease
MRI: Magnetic resonance imaging
NTR: Near total resection
NSCLC: Non-small cell lung cancer
OS: Overall survival
PT: Primary Tumor
RT: Radiation Therapy
SRS: Stereotactic radiosurgery
STR: Subtotal resection
WBRT: Whole brain radiation therapy
