# Supplementary figures and images for: Leptomeningeal disease (LMD) after resection of brain metastases: results of the multicenter SUBAROMA study
Source: J Neurooncol. 2026 Jul 10;178(3):99. doi: 10.1007/s11060-026-05700-6 (PMC13354617; doi:10.1007/s11060-026-05700-6)

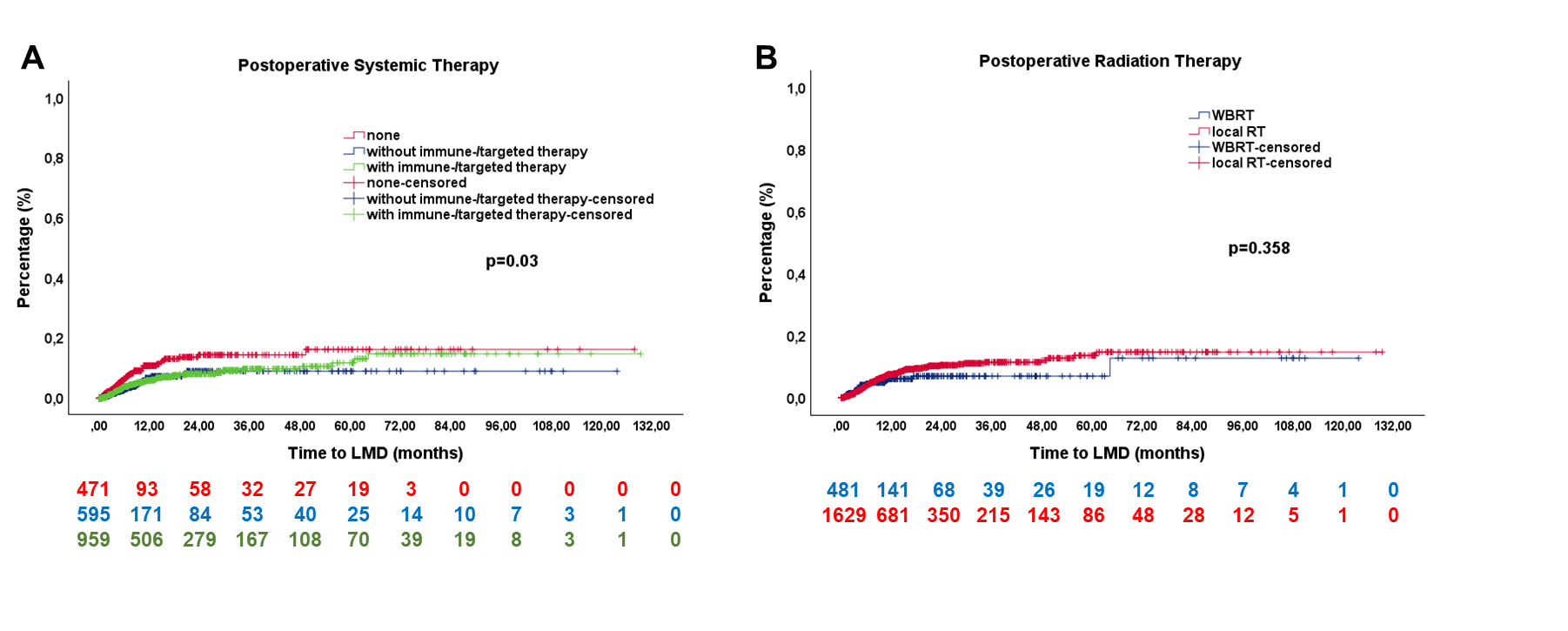

Supplement: Supplementary file 1 — Supplementary Material 1 [file 11060_2026_5700_MOESM1_ESM.tif]
