# Supplementary material for: Leptomeningeal disease (LMD) after resection of brain metastases: results of the multicenter SUBAROMA study
Source: J Neurooncol. 2026 Jul 10;178(3):99. doi: 10.1007/s11060-026-05700-6 (PMC13354617; doi:10.1007/s11060-026-05700-6)
Supplement: Supplementary file 2 — Supplementary Material 2 [file 11060_2026_5700_MOESM2_ESM.docx]

Supplement 1. Kaplan-Meier-Analyses for risk factors for LMD progression associated with therapy: (A) systemic therapy, (B) radiation therapy; WBRT: whole brain radiation therapy RT: radiation therapy.
